# Supplementary figures and images for: CBFB cooperates with p53 to maintain TAp73 expression and suppress breast cancer
Source: PLoS Genet. 2021 May 4;17(5):e1009553. doi: 10.1371/journal.pgen.1009553 (PMC8121313; doi:10.1371/journal.pgen.1009553)

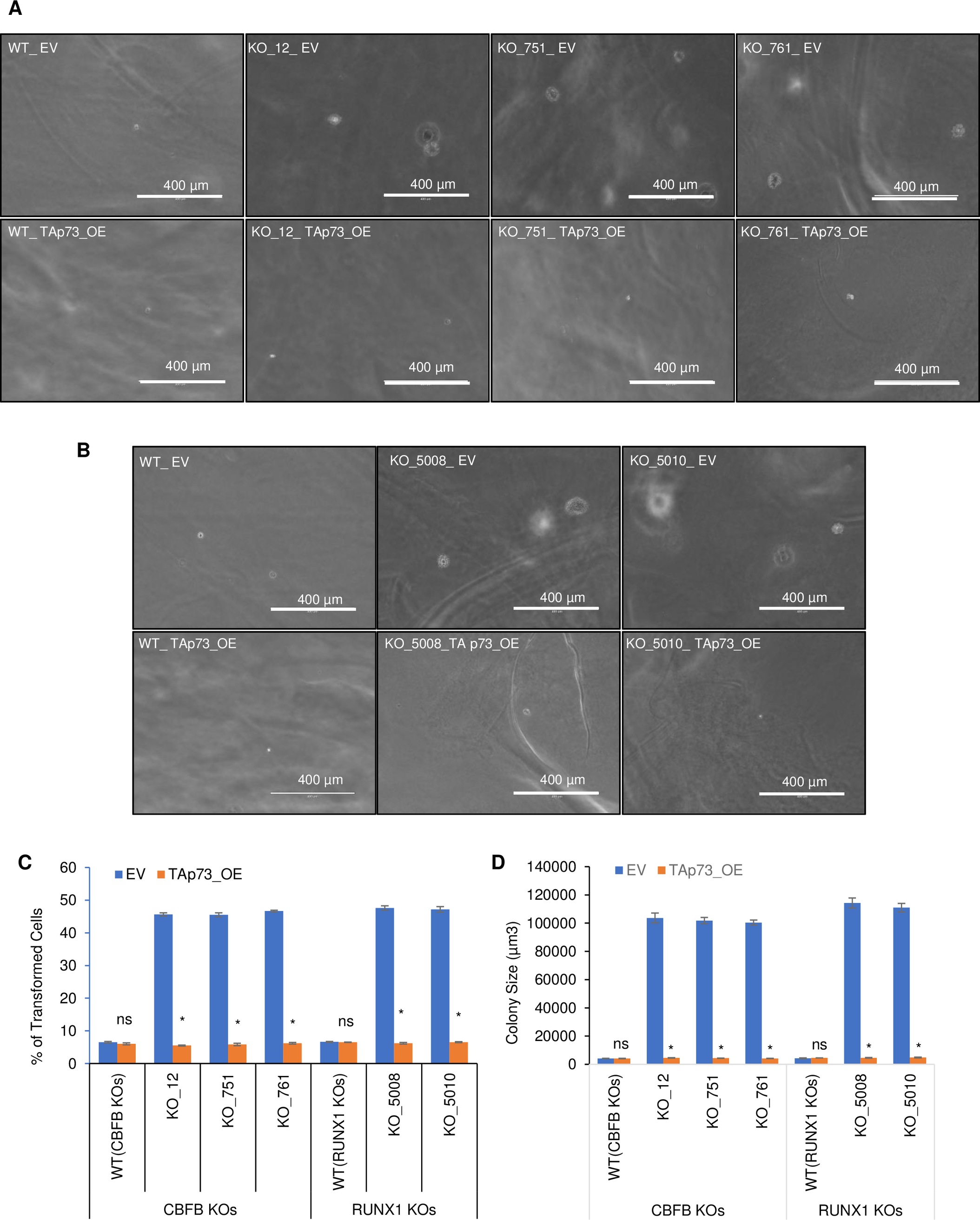

Supplement: S1 Fig — (A & B) Representative images of anchorage-independent growth assays to show the effect of exogenously expressed TAp73 (p73_OE) on colony formation in CBFB_KO (A) and RUNX1_KO cells (B). C, Percentage of transformed cells, as evaluated by the number of colonies formed, in CBFB_KO and RUNX1_KO cells after overexpression of TAp73, 30 days after plating. Error bars are SEM, n = 3 (biological repeats); one asterisks, p-value <0.01, ns, p-value >0.05 (empty vector, EV versus TAp73 OE). The t-test is two-tailed, two-sample equal variance. D, Size of colonies in CBFB_KO and RUNX1_KO cells after overexpression of TAp73, 30 days after plating. Error bars are in SEM, n = 3 (biological repeats); one asterisks, p-value <0.01; ns, p-value >0.05 (EV versus TAp73 OE). The t-test is two-tailed, two-sample equal variance. (TIF) [file pgen.1009553.s001.tif]

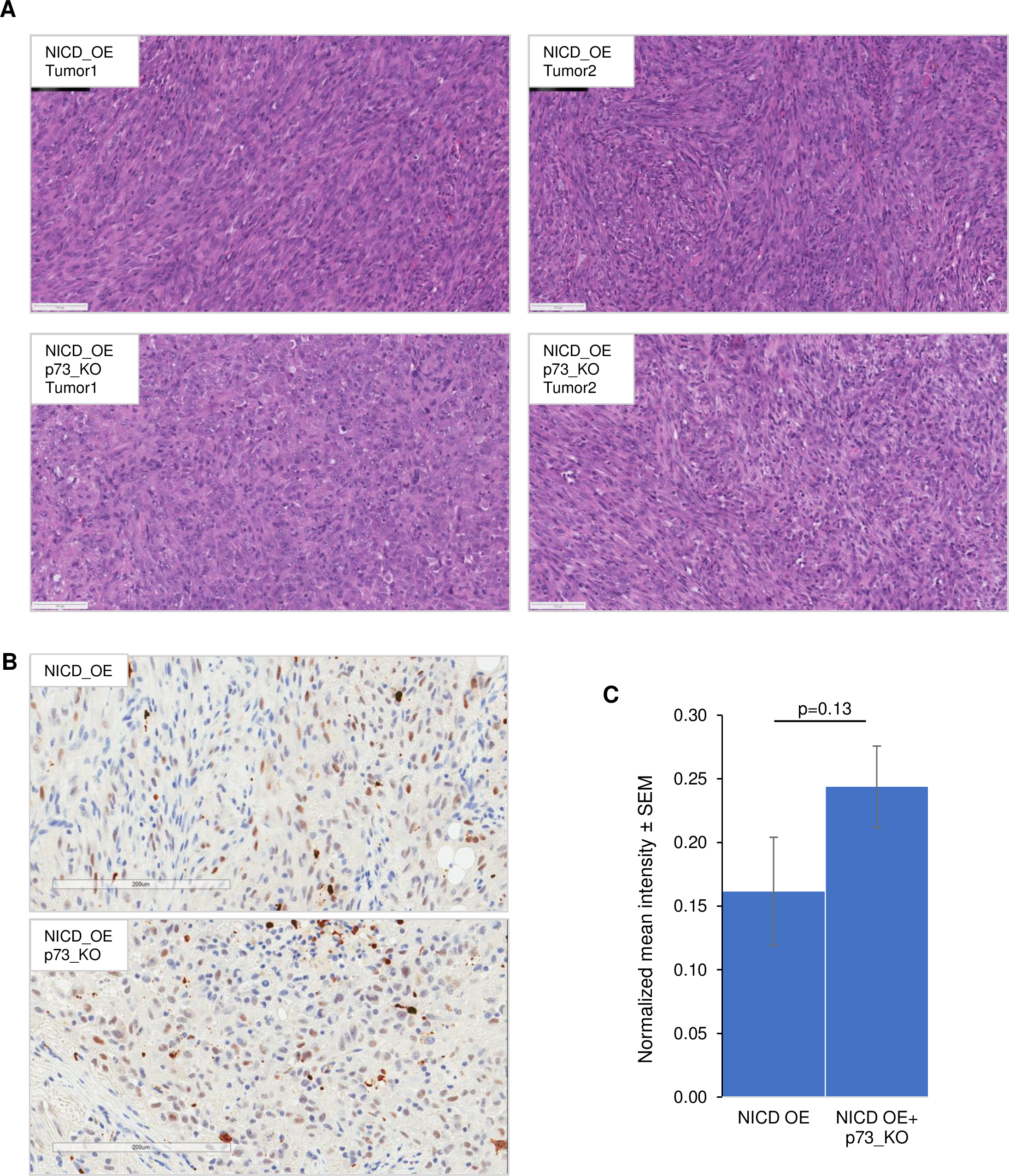

Supplement: S2 Fig — (A) H&E staining. Representative images from two tumors were shown. Scale bar, 100 μm. (B) IHC of cleaved caspase 3. Scale bar, 200 μm. (C) Normalized mean intensity of cleaved caspase 3. See Materials and Methods for calculation of normalized mean intensity. Shown is the average of normalized mean intensity ± SEM from 15 images (5 randomly selected images from each of the three tumors). P-value is from t-test (two-tailed, two-sample equal variance). (TIF) [file pgen.1009553.s002.tif]

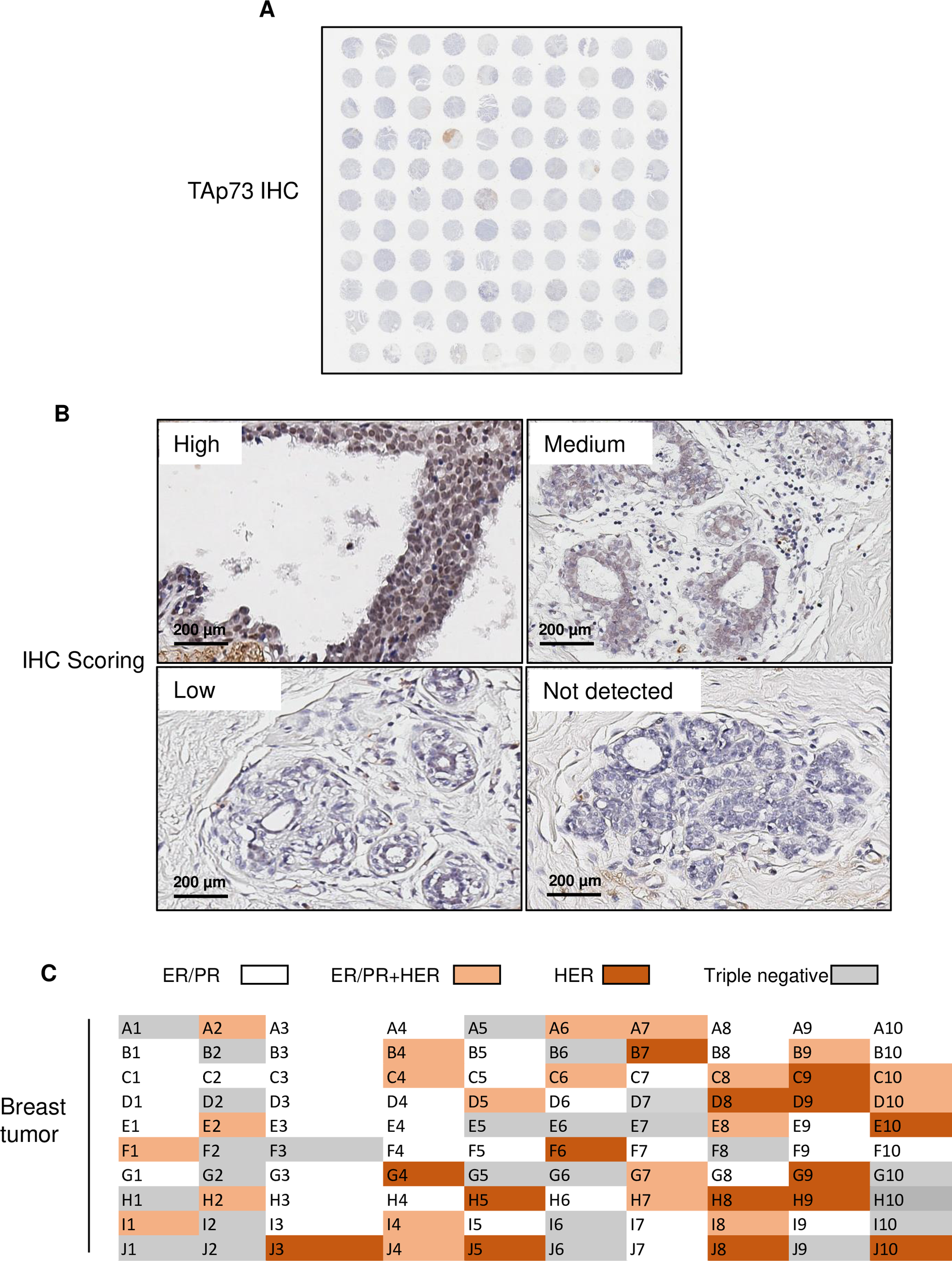

Supplement: S3 Fig — (A) TAp73 IHC images in a human breast tumor tissue microarray (TMA), which includes 10 normal breast tissue samples at the bottom. (B) Scoring examples of TAp73 IHC staining for high, medium, low, and not detected in TMA. (C) Subtypes of breast tumor samples in the TMA: Estrogen receptor (ER)/Progesterone receptor (PR) positive, ER/PR, and HER2 receptor-positive, HER2 receptor-positive and triple-negative. (TIF) [file pgen.1009553.s003.tif]
