# Supplementary material for: CBFB cooperates with p53 to maintain TAp73 expression and suppress breast cancer
Source: PLoS Genet. 2021 May 4;17(5):e1009553. doi: 10.1371/journal.pgen.1009553 (PMC8121313; doi:10.1371/journal.pgen.1009553)
Supplement: Attachment — Submitted filename: responses.docx [file pgen.1009553.s005.docx]

We thank the reviewers for considering our study interesting and original. We are very grateful to our reviewers for their constructive comments and suggestions, which have significantly improved our manuscript. Our point-by-point responses are as follows. Please be advised that all the major changes are highlighted in yellow in the manuscript text.

**Reviewer #1:**

“*Overall experiments are well designed and results support the major conclusions.*”

Response: We thank the reviewer for the kind words.

Some minor points:

“*1. Fig 1F: it appears that p53 KO slightly reduces the CBFB levels. Authors may want to*

*confirm this result or modify their statement that p53 does not affect CBFB expression in*

*breast cell lines.*”

Response: The reviewer raised a good point. The increase of CBFB in p53 KO is consistent but subtle. Therefore, we have modified our statement, as suggested by the reviewer. In addition, normalized (to β-actin) values of CBFB were shown in this submission.

“*2. Authors found that p53 and CBFB can regulate a set of genes in the same direction. p73 was*

*proved to be an important downstream gene that mediates CBFB function in tumor suppression in breast cancer. Authors may want to discuss whether it is possible that some other genes in their list also contribute to the function of CBFB in breast cancer.*”

Response: We agree with the reviewer. It is possible that other common targets also partially mediate the tumor suppressive role of CBFB in breast cancer. We added several sentences to highlight this point in the Discussion section.

“*3. As authors described, p73 expression is frequently lost in breast cancer. It will be nice if*

*authors can discuss other mechanisms contributing to p73 loss in breast cancer in addition to*

*CBFB mutations given that CBFB mutations are not very common in breast cancer (around*

*5%).*”

Response: It is true that other mechanisms contribute to p73 loss. Thus, we added discussion on this point in the Discussion section (At the end of the 2^nd^ paragraph).

**Reviewer #2:**

“*1) As authors may be aware, mutual exclusivity of two gene mutations in cancer may result*

*from cancer cell death that is caused by co-occurrence of two gene mutations. Several*

*examples are described in following articles: 1) Cisowski and Bergo, 2017 [PMID:*

*27416373]; 2) Zhao et al 2017 Nature [PMID: 28166537]). In this regard, although it is*

*plausible that mutual exclusivity of CBFB and TP53 mutations would implicate a functional*

*link between CBFB and TP53, it would not be reasonable to mention that the co-activation of*

*the tumor suppressor gene TAp73 by CBFB and p53 would provide a mechanistic explanation*

*to the mutual exclusivity of CBFB and TP53 mutations. This is because TAp73*

*downregulation that is caused by CBFB or TP53 depletion did not result in cancer cell death*

*but increased tumorigenicity of NOTCH3-overexpressed MCF10A cells.*

*Therefore, they need to reword (or delete) multiple sentences throughout the text. Examples*

*include but not are limited to the following ones: Abstract (therefore providing a mechanistic*

*explanation to the mutual exclusivity of CBFB and TP53 mutations, page 2); discussion (This*

*model explains the mutual exclusivity of CBFB and TP53 mutations, page 15); and Fig 7*

*legend (This model can explain the mutual exclusivity of CBFB and TP53 mutations in breast*

*tumors).. In addition, the bottom panel in Fig. 2A may not be needed.*”

Response: We agree with the reviewer and removed/reworded sentences similar to “therefore providing a mechanistic explanation to the mutual exclusivity of CBFB and TP53 mutations” throughout the manuscript*.* In addition, we removed Fig. 2A since it was described in the main text.

“*2) In abstract, authors mention “a functional interaction between CBFB and p53”. In addition,*

*authors mention “suggesting that CBFB functionally interacts with p53 in breast cancer cells”*

*in page 6. There are similar statements. “Functional interaction” and “functionally interacts”*

*might be too specific. Authors should use a different word.*”

Response: The reviewer is correct. In this submission, we replaced “functional interaction” and “functionally interacts” with less specific words, such as “functional communication”, “functional association”, “communication”, “crosstalk”, or “functionally converge”.

“*3) In abstract, authors introduce NOTCH3 without an explanation. It would be helpful for*

*readers if they mention like “overexpression of NOTCH3, a CBFB-suppressed oncogene”*

*instead of “NOTCH3 overexpression*”

Response: We thank the reviewer for this excellent suggestion, which increases the clarity. We have modified the abstract accordingly.

“*4) In Fig. 2D, where are the p53’s consensus binding sequences? Please mark them.*”

Response: We marked the p53 consensus motif. Please be advised that this is a degenerated motif, which is 96-bp away from the RUNX1 motifs.

“*5) ChIP-seq data for p53 and RUNX1 are good results. To examine whether p53 and*

*RUNX1/CBFB cooperate to regulate TAp73, authors need to show a more convincing result.*

*Does p53 activate the FL-RE reporter (relevant to Fig. 2E)? Does the co-transfection of p53 and RUNX1 (or CBFB) activate reporter gene expression more than single transfection?*”

Response: To answer these questions, we performed additional reporter assays. The data were shown in new Fig 2H. p53 activated the FL-RE reporter. Overexpression of CBFB or p53 enhanced RUNX1-driven reporter activity.

“*6) Authors need to show data (that may be similar to the bottom panels in Fig. 1A & 1B) for*

*epistasis analysis in ER-positive breast tumors.*”

Response: In this submission, we provided the data in new Fig. 1C.

“*7) In Fig 3B-3H, there are numbers (e.g., 12, 751 and 761) as labels. Although these numbers*

*look clone numbers, authors should clearly indicate what these are in legends or figures.*”

Response: We apologize for this oversight. In this submission, we indicate that these numbers are clone IDs in the figures.

“*8) It seems unusual to use TS for tumor suppressive.*”

Response: We have replaced all “TS” with “tumor suppressive”.

“*9) In page 11, the reduction TAp73 expression upon CBFB or RUNX1 deletion would better*

*be “the reduced TAp73 expression….” or “the reduction in TAp73 expression….”*”

Response: We thank the reviewer for pointing out this grammar error, which was corrected in this submission.

**Reviewer #3:**

“*In this manuscript, the authors unmask a novel tumour suppressor mechanism in*

*breast cancer, which is built around the CBFB-TAp73 axis. The authors find that CBFB and*

*TP53 mutations are mutually exclusive in breast cancer. Further analysis suggest that p53 and*

*CBFB converge towards a common regulation of the TAp73 gene. Indeed, CBFB maintain*

*TAp73 expression in the MCF10A breast cells. TAp73 regulation has important functional*

*implications. Indeed, loss of TAp73 cooperates with NOTCH3 overexpression in augment if*

*breast cancer cell tumorigenicity in vitro and in vivo. A tumour suppressive role for TAp73 is*

*confirmed by its loss of expression in a subset of breast cancers.*

*I found this paper to be well-written and quite interesting to read. Some of the findings are*

*intriguing and of potential relevance for the scientific community. I have few observations,*

*which I hope the authors will find helpful.*”

Response: We are very grateful to the reviewer for the encouraging words and helpful suggestions. Our responses are as follows.

“*Initially the authors exclude any direct regulation between p53 and CBFB. They support this*

*conclusion with the western blot presented in Figure E and F. I assume it will not come as a*

*surprise to the authors if I do not fully agree with their interpretation. Indeed, it seems that*

*CBFB levels are increased in p53 KO clones. I would appreciate if the authors could reassess*

*this experiment. Perhaps a quantification of the western blot bands (using a less exposed actin)*

*could be informative. To be honest, even if my observation of the data should turn out to be*

*correct, I do not feel that this would detract from the strengths of the manuscript and I would*

*still endorse its publication. It might however entail some degree of rewriting*”

Response: The reviewer is correct. We examined our other data and consistently observed a slight increase of CBFB in p53_KO MCF10A cells. After normalizing to the β-actin signal, the increase was about 15%, indicated in Fig 1F. As the reviewer suggested, we reworded our interpretation (see highlight in the text).

“*I was also wondering why the authors observe two bands for CBFB protein in panel F and a single band in panel E.*”

Response: Our laboratory has been using the NuPAGE gel system (Life Technologies), and it has two buffers: the MOPS and MES buffers. MOPS is suitable for separating proteins with middle or large molecular weight, while MES for proteins with small molecular weight. CBFB is about 20 Kilo Dalton. Therefore, we often use the MES buffer and generally observe two bands. Sometimes, we use MOPS buffer to favor the separation of proteins with middle or large molecular weight. In this case, the two bands for CBFB are often compressed into one.

“*I shall confess that this reviewer is not an expert of ChIP-seq. However, at least from the data*

*presented in figure 3A, it seems that the H3K4me3 signals is decreased in the DNp73*

*promoter in p53 KO cells, which would contradict the authors’ interpretation of the results. I*

*would appreciate if the authors could comment on this. Have the authors picked any signal*

*from the H3K4me3 in the intragenic region that harbours the RUNX1 binding sites?*”

Response: The reviewer is correct that the H3K4me3 signal in the DNp73 promoter in p53_KO cells was slightly reduced. This comment is important and touches on a fundamental question of histone modifications: what are their functions? The answer is unclear. Changes of H3K4me3 may or may not correlate with gene expression changes. A classical study of H3K4me3 in mammalian cells (*Cell*, Vol:144, 513-525, 2011) from Dr. Robert Roeder’s group showed that reduced H3K4me3 signal correlates with reduced induction of some developmental genes (Fig. 4 in the paper) but not p53-induced p21 and Mdm2 (Fig S3 in the paper). Therefore, a reduction in H3K4me3 is only suggestive, not definitive, that p53, CBFB, or RUNX1 regulates the expression of a specific gene. Gene expression experiments are needed. In our study, we used immunoblotting. To increase clarity, we included this observation (highlighted in yellow) in our manuscript.

It is generally true that H3K4me3 marks the promoter regions. Therefore, our main purpose in using H3K4me3 is to identify the promoters of *TAp73* and *DNp73*. After combining with gene annotation, we are confident with the identified promoters. However, due to the chromatin looping, H3K4me3 can be detected in other regions, such as enhancers, and within the gene body. We did observe a small peak in the intragenic region containing the RUNX1 binding sites, probably due to the looping of this intragenic region with the promoter.

“*The luciferase experiments are quite convincing and show a thorough analysis of the RUNX1*

*bindings sites, although I would argue that site 1 seems to be quite irrelevant compared to the*

*impact of mutating the site 2 and 3. Perhaps statistical analysis of the Luc experiments could*

*help discerning the impact of the different site mutation, especially site 1.*”

Response: The contribution of site 1 is more complicated than those of sites 2 and 3. On the one hand, mutating site 1 alone did not alter the luciferase activity, as the reviewer pointed out. On the other hand, in combination with either site 2 or 3, the effect of site 1 became prominent. Please compare the luciferase activity of Mut1 to control, Mut1_2 to Mut2, Mut1_3 to Mut3, or Mut1_2_3 to Mut2_3. As suggested, we added statistical analyses on Fig. 2G. In addition, we modified our text to add clarity (see highlight in yellow).

“*In addition, I was wondering whether the authors could ascertain if the response of the luciferase construct to RUNX1 is abolished in CBFB KO cells. I am asking this on the light of the data reported in Figure 3H, where RUNX1 overexpression restores TAp73 levels in CBFB KO cells, indicating that RUNX1 might regulate p73 expression at least partially independently of CBFB.*”

Response: The reviewer raised an important point. To answer this question, we performed luciferase assays in WT and CBFB_KO cells (new Fig. 2E). Loss of CBFB decreased the response of the reporter to RUNX1. However, RUNX1 still induced the reporter in CBFB_KO cells. Our interpretation is that CBFB deletion does reduce RUNX1 activity in p73 induction. But when RUNX1 is over-expressed, it can overcome the effect of CBFB loss. This result is consistent with the model that CBFB is a facilitator but not an on-off switch for RUNX1’s transcriptional activity.

“*The xenograft experiments are interesting. I was hoping that the authors could provide an*

*H&E assessment of those tumours. It would be interesting to see if loss of p73 alters tumours*

*histology. Similarly, it would be nice, though not essential, if the authors could assess*

*expression of ki67 and cleaved caspase 3 by IHC in those tumours as an indication as to*

*whether p73 loss affect proliferation/survival.*”

Response: We thank the reviewer for suggesting these informative experiments. In this submission, we performed H&E staining and did not detect a noticeable difference in histology between NICD OE and p73_KO+NICD OE tumors (S2A Fig). Interestingly, the Ki-67 signal in p73_KO+NICD OE tumors was about 5 times higher than that in NICD OE tumors (new Fig 5K-L). This result suggests that p73 loss promotes tumor cell proliferation, consistent with the observation that tumor size and weight in the p73_KO+NICD OE group are bigger than those of the WT group. We did not detect a statistically significant difference in cleaved caspase 3 between the two groups (S2B-C Fig).

“*With regard to loss of TAp73 in human breast cancer, I am well aware of the suboptimal*

*performances of most antibodies against p73 proteins. However, it would important to provide*

*clear example images of the scoring presented in Figure 6D (high, medium, low…).*”

Response: In this submission, we provided example images for scoring in S3B Fig.

“*Finally, would it be possible for the authors to retrieve information about TAp73 expression in the breast cancer datasets and compare expression levels in CBFB/p53 wild-type vs CBFB*

*mutated tumours? I appreciate this might not be possible, but it could provide a nice*

*confirmation of the interaction between CBFB and TAp73 in human samples.*”

Response: This is a good suggestion. We were able to examine the expression of TAp73 in the TCGA dataset. Tumors were categorized into four groups: TP53_MUT;CBFB_MUT, TP53_MUT;CBFB_WT, TP53_WT;CBFB_MUT, and TP53_WT;CBFB_WT. TAp73 levels were significantly lower (p=7.8e-08) in the TP53_MUT;CBFB_WT group (median=19.93) than the TP53_WT;CBFB_WT group (median=57.59). TAp73 levels in the TP53_MUT;CBFB_MUT group (median=43.85) and the TP53_WT;CBFB_MUT (median=28.24) were lower than that in the TP53_WT;CBFB_WT group (median=57.57). However, the differences were not statistically significant, which may be due to 1) the small number of tumors for these two groups (3 for TP53_MUT;CBFB_MUT and 20 for TP53_WT;CBFB_MUT). Unfortunately, the expression data in the METABRIC dataset was generated with microarrays. So, there is no isoform information for this dataset. 2) the heterogeneity of tumors. We do not know whether TP53 and CBFB mutations affect the tumor microenvironment, such as stromal or immune cell infiltration. Therefore, RNA-seq data may be “contaminated” by infiltrated stromal and immune cells.

“*The p73KO cells seem to be knockout for the whole gene and not selective for TAp73.*

*Perhaps the authors could elaborate on this understandable limitation in the discussion section.*”

Response: The reviewer is correct. Our p73_KO cells are not selective for TAp73. As suggested by the reviewer, we pointed out this caveat in the Discussion section to alert the readers.

“*The author should expand the materials and methods section providing a paragraph detailing*

*the statistical analysis approached used in the manuscript. Similarly, an accurate description of*

*the methodology used for the TMA scoring is necessary.*”

Response: We have added one paragraph in the Materials and Methods section to describe statistical analyses and TMA scoring method.

MINOR POINTS

“*I would appreciate that, if this is in agreement with the journal guidelines, the authors could*

*explain figure legends to include details of descriptive and inferential statistical analysis for*

*the different graph/data reported.*”

Response: In this submission, we added a description of statistical analysis in the Legends.

“*I would also be grateful if the authors could indicate the bench mark molecular weight in the*

*western blots.*”

Response: In this version, we have added the molecular weight of the protein ladder. Please be advised that the marker we use is the SeeBlue marker (Life Technologies). The apparent molecular weight of the proteins in the ladder in the NuPAGE gel system is different from that in the Tris-Glycine using the SDS Running Buffer. We described this in the Materials and Methods section.

“*Honestly, I do not see the point of Figure 1D and Figure 2A. The author’s hypotheses, in both*

*cases, are clearly outlined in the main text.*”

Response: As suggested, we removed Fig 1D and 2A in this submission.

“*The description of the generation of p73KO cells seems to have been written twice in the*

*methods section, with a minor change in one of the gRNA sequence. Could the author please*

*check this?*”

Response: We apologize for the redundancy. In this submission, we removed the wordy sentences and verified the sequence of gRNAs.

“*One aspect that I like about this manuscript is that it describes TAp73 in its “original role” as*

*tumour suppressor gene. Indeed, this is in contrast with recent reports indicating that TAp73*

*could paradoxically sustain tumour progression, especially through regulation of tumour*

*cellular metabolism. Notably, this has also been reported for breast cancer stem cells. It would*

*be interesting if the authors could comment their results on the light of these works. Indeed,*

*their observations would suggest that the impact of TAp73 expression could depend on the*

*subtype of breast cancer, with CBFB/NOTCH3-driven cancer highlighting a TS function for*

*the TAp73 gene. However, although intrigued, I would understand if the authors should feel*

*that this discussion might not be relevant to their analysis.*”

Response: We agree with the reviewer that there are several reports showing TAp73 could sustain tumor progression via its role in metabolism. For example, TAp73 has been shown to regulate glutamine addiction in medulloblastoma (Niklison-Chirou et al., *Genes and Dev* 2017), serine metabolism in lung cancer and osteosarcoma cells (Amelio et al., *Oncogene* 2013). As pointed out by the reviewer, a study using embryonal carcinoma stem-like cells and breast cancer stem cell-like cells (Sharif et al., *Clinical Cancer Research* 2018) showed that TAp73 is critical for the self-renewal, tumorigenicity, redox balance, and metabolism of these cells. Interestingly, the authors in this paper mentioned that the roles of TAp73 in their stem-like cells are different from those in well-differentiated cancer cells. Indeed, our study focuses on differentiated cells. Therefore, it is likely that the roles of TAp73 depend on cancer types and specific populations within a given cancer type. Or, as the reviewer alluded, the unique genetic makeup of a tumor cell could alter the role of TAp73.

To alert the readers, we added several sentences in the Introduction and Discussion sections to point out these studies.
